# Stem cell fate decisions: Substates and attractors

**DOI:** 10.1016/j.stemcr.2025.102532

**Published:** 2025-06-10

**Authors:** James E. Mason, Xiaokai Nie, Daniel Coca, Peter W. Andrews

**Affiliations:** 1 The School of Biosciences, The University of Sheffield, Western Bank, S10 2TN Sheffield, UK; 2 School of Electrical and Electronic Engineering, The University of Sheffield, Western Bank, S10 2TN Sheffield, UK; 3 School of Engineering, Newcastle University, NE1 7RU Newcastle-upon-Tyne, UK

**Keywords:** human, pluripotent stem cells, differentiation, substates, deterministic chaos

## Abstract

Heterogeneity of gene expression is a characteristic of stem cell populations. Here, we review our own findings with human pluripotent stem cells, emphasizing their capacity to occupy distinct metastable substates that influence their differentiation outcomes. Experimental studies, such as single-cell transcriptomics and analyses of marker dynamics, demonstrate the transient and dynamic nature of these substates. Based on a novel approach to model sequences of fluorescent marker distributions over time, we suggest that transitions between these substates may be governed by deterministic chaos in addition to stochastic fluctuations of gene expression, both of which likely contribute to the observed dynamic behavior.

## Graphical abstract

**Figure:**
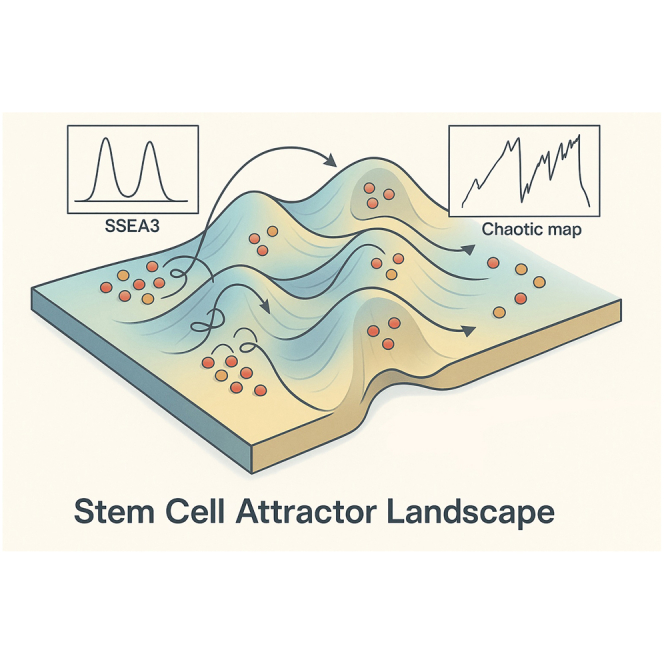

## Teaser

In this short perspective, the authors have summarized their evidence for lineage-biased metastable substates of pluripotent stem cells and mathematical tools that can be used to analyze the dynamics of their interconversion, which may be governed by deterministic chaos.

## Introduction

Central to the biology of multicellular organisms are the mechanisms by which a single cell multiplies and gives rise to other cells with diverse phenotypes, both during embryogenesis and in tissue turnover and repair in the adult. Disruption of these processes is fundamental to cancer formation. It is well established that cell differentiation primarily involves differential expression of the gene set required to specify the whole organism. Much analysis focuses on the role of key gene regulatory networks (GRNs) that, once set up in response to external signals, are self-maintaining until disturbed by further signals. This has provided important insights into the mechanisms by which pluripotent stem cells (PSCs) maintain their pluripotent state or initiate pathways of differentiation that lead to the appearance of multiple cell types during embryogenesis (Smith 2024). However, gene expression regulation is typically considered in terms of simple on/off switches, overlooking the potential for variable levels of gene activity or considering the myriad of other factors resulting from the metabolism of the cell and the laws of mass action that might affect the probability of specific events occurring in the genome at specific times. Given the vast array of signals and events that might impinge on the behavior and outcomes of particular sets of gene activity, an alternative approach is to view cells as dynamical systems, considering their behavior holistically without focusing on the fine-grained details of the many interacting factors influencing a cell’s state at a particular time. Building on Waddington’s ideas (Waddington 1957), which resonate with thermodynamic concepts (Goodwin 1963), specific cell types may be considered as stable states, or attractors, within a landscape that comprises all possible states of the system (Kauffman 1967; 1993). In this context, an “attractor” represents a stable, self-sustaining gene expression pattern corresponding to a specific cell type or fate. Differentiation, where PSCs evolve from a pluripotent state toward specialized cell fates, can be conceptualized as transitions between attractor states. The landscape reflects the likelihood of these transitions between states (Enver et al., 2009).

## The attractor hypothesis and its mathematical foundations

The mathematical foundations for the attractor hypothesis (Huang et al., 2005; Huang, 2009; Kauffman, 1993) provide a framework for quantitatively understanding how PSCs maintain their pluripotent state or transition to specific cell lineages. An attractor of a dynamical system is a set of states toward which trajectories of the system starting in a local region evolve. In the context of PSCs and GRNs, attractors represent stable patterns of gene expression corresponding to specific cell types or fates. Each gene’s activity or expression level can be represented as a state variable, denoted as $x_{i}$, that quantifies the expression level of the gene. The expression levels of all genes in the GRN collectively form a high-dimensional vector representing the cell state (Huang et al., 2005):

(Equation 1) $$x=\left(x_{1},x_{2},\ldots ,x_{N}\right)$$

where $x_{i}$ is the expression level of the *i*-th gene and *N* is the number of genes. The cell state vector captures the complete expression profile of the GRN at a specific point in time.

The temporal evolution of the system can be described by differential equations:

(Equation 2) $$\frac{dx}{dt}=F\left(x_{1},x_{2},\ldots ,x_{N}\;\right)$$

where $F\left(x\right)=\left(f_{1}\left(x\right),f_{2}\left(x\right),\ldots ,f_{N}\left(x\right)\right)$ describes the typically nonlinear interactions among genes i.e., activation or repression, negative or positive feedback, or cooperative binding interactions.

Mathematically, for a dynamical system (2), an attractor set *A* is such that if x(t)∈A at a time t^∗^ then x(t)∈A for t>t^∗^, that is, for all the future evolution of the system. Attractor sets can be classified into *point attractors*, where gene expression levels stabilize at constant values over time; *stable limit cycles*, which correspond to cyclical cellular processes; and *strange or chaotic attractors*, which exhibit a fractal structure, where dynamics appear random but are deterministic. In the context of cell differentiation, a stable cell fate may be characterized by a combination of point attractors for some genes and stable limit cycles, where the expression of specific genes, such as those involved in circadian rhythms or the cell cycle, oscillates periodically to support essential cellular functions without altering the overall identity of the cell.

**Figure 1:**
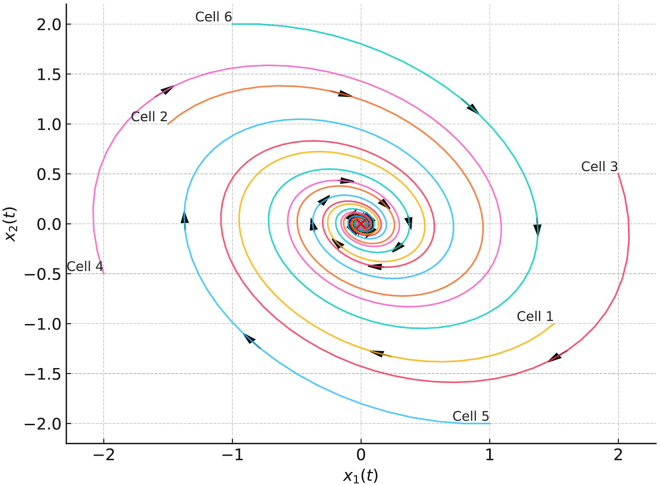
Illustration of gene expression trajectories of cells starting from different states as they move toward a stable attractor, corresponding to a specific cell fate In this diagram, x1(t) and x2(t) represent expression levels for two genes at time t. Trajectories show the evolution of a cell’s gene expression state over time, highlighting how different initial states follow distinct paths but ultimately converge to the stable equilibrium point representing a distinct cell fate.

The region in the state space from which trajectories converge to an attractor is known as the basin of attraction for that attractor. In the context of GRNs, the basin of attraction represents the range of gene expression patterns that guide a cell toward a specific fate. Figure 1 illustrates a two-dimensional representation of trajectories of gene expression levels over time for different initial cell states within the basin of attraction, where each cell follows a dynamic path toward a stable attractor.

## Stochastic dynamics, noise-induced transitions, and deterministic chaos

Inherent fluctuations in gene expression due to the probabilistic nature of molecular interactions can induce transitions between attractor states (Chambers et al., 2007). The size and “depth” of a basin of attraction are indicative of a cell fate’s robustness: larger, “deeper” basins indicate a more stable cell fate, where cells are resilient to small perturbations and will maintain their identity even with minor changes in conditions.

Mathematically this can be described by a stochastic differential equation (Zhou et al., 2012)

(Equation 3) $$\frac{dx}{dt}=-\nabla U\left(x_{1},x_{2},\ldots ,x_{N}\;\right)+\eta \left(t\right)$$

where ∇U is the gradient of the potential function that drives the system toward a stable state and η(t) denotes the noise that potentially causes transitions between attractor states. Although a potential function provides a useful framework for describing state transitions in certain stochastic systems, it is not a general property of all dynamical systems, particularly GRNs. In many cases, the complexity and feedback dynamics of GRNs prevent a straightforward potential function representation. However, quasi-potential approximations can still offer insights into stability and transition dynamics in PSC populations.

PSCs exhibit intrinsic instability, as evidenced by their tendencies toward spontaneous differentiation and heterogeneity of gene expression patterns. One possibility is that the basins of attraction for PSC stable states are shallow, making PSCs highly susceptible to minor perturbations that prevent most cells from settling into a defined identity.

Alternatively, this instability could be interpreted through a chaotic hypothesis (Furusawa and Kaneko, 2009), proposing that cellular fates arise from a dynamically unstable system in which gene expression levels fluctuate within a high-dimensional chaotic attractor. In this framework, transitions between quasi-stable states are driven by transient chaotic dynamics, enabling cells to navigate and explore multiple potential fates. This perspective aligns with emerging views in cancer biology, for example, which attribute the heterogeneity of glioblastoma stem-like cell populations and their resilience to external perturbations, such as therapeutic interventions, to chaotic state fluctuations within a perturbed genetic landscape (Prager et al., 2020). This dynamic behavior allows cells to simultaneously occupy a multitude of substates, contributing to their adaptability and persistence.

## Substates of pluripotent stem cells—Empirical insights

The concept of cellular metastability, where cell populations fluctuate between quasi-stable states, is increasingly recognized as a fundamental property of stem cells. In this respect, Chang et al. (2008) observed quasi-stable behavior in subpopulations of hematopoietic progenitor cells, which are consistent with the notion of cellular metastability. Specifically, they found that spontaneous “outlier” cells with either extremely high or low expression levels of the stem cell marker Sca-1 eventually revert to the parental Sca-1 distribution after more than a week. These findings suggested that heterogeneity in gene expression is not simply the result of independent noise in individual gene expression, but rather reflects metastable states, or transient attractors, explored by a slowly fluctuating transcriptome, which allows multipotent progenitor cells to remain primed yet flexible to preserve their potential for differentiation.

Our initial indication that cultures of human PSCs might contain cells that exist in interchangeable substates came from a comparison of the transcriptome of a karyotypically normal human embryonic stem (ES) cell line, H7, and a karyotypically abnormal variant that we designated culture adapted (Enver et al., 2005). Since human ES cultures often contain “contaminating” differentiated derivatives, we used the surface marker SSEA3 to isolate the undifferentiated cells of these lines for transcriptomic analysis. SSEA3 is a well-established marker of undifferentiated human PSCs but is notable for its relatively rapid disappearance early in a pathway of differentiation (Fenderson et al., 1987). In the karyotypically normal cultures, as expected, the clonogenic cells were almost exclusively found among the SSEA3(+) cells. However, to our surprise, in cultures of the karyotypically abnormal line, we found clonogenic cells in both the SSEA3(+) and SSEA3(−) subpopulations. This led us to suggest that the karyotypically normal ES cells oscillate between SSEA3(+) and SSEA3(−) substates but that cells in the SSEA3(−) substate have a high probability of differentiating and leaving the stem cell compartment so they can rarely be detected. By contrast, we posited that the karyotypically abnormal cells have a block reducing their probability of differentiation, so that cells in the SSEA3(−) substate are trapped within the stem cell compartment and can be readily detected. Studying a different human ES cell line and two different developmentally regulated cell surface marker antigens, GCTM2 and CD9, Laslett and colleagues also came to the conclusion that undifferentiated PSCs exist in a hierarchy of substates comprising the stem cell compartment before differentiation (Laslett et al., 2007).

Although the expression of SSEA3, GCTM2, and CD9 correlates with the undifferentiated state of human PSCs, their functional role, if any, in maintaining that state is obscure. However, another study of the transcription factor NANOG also indicated the existence of substates in the stem cell compartment of mouse ES cells (Chambers et al., 2007). NANOG was initially thought to be required to establish and maintain the pluripotent state. However, the study of Chambers and colleagues showed that mouse PSCs maintained pluripotency in the absence of NANOG but that these NANOG(−) cells were unstable and tended to differentiate rapidly, a condition paralleling the proposed behavior of SSEA3(−) human PSCs. In human PSCs, the expression of NANOG is dependent on extracellular activin signaling, which in turn is dependent on an intracellular signaling pathway involving SMAD4. Intriguingly, we found that human ES cells in which the expression of SMAD4 is conditionally repressed remained in a pluripotent stem cell state, but this state is unstable and particularly susceptible to differentiation (Avery et al., 2010).

**Figure 2:**
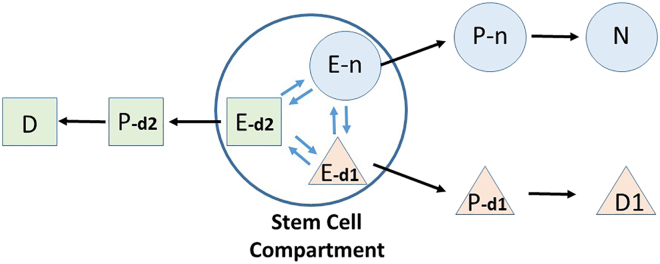
Schematic of neuronal and non-neuronal-biased substates of undifferentiated NTERA2 stem cells In this model, the undifferentiated stem cells exist in different substates, here illustrated as E-n, E-d1, and E-d2, which readily interconvert. Together, these different substates comprise the “stem cell compartment.” When induced to differentiate by, for example, retinoic acid, cells in each substate give rise to progenitor cells of distinct lineages, P-n, P-d1, and P-d2, which may subsequently proliferate before final differentiation into terminal cell types such as neurons, N, and other non-neuronal differentiated cells, D1 and D2. Thus, if a single cell is isolated from the stem cell compartment and allowed to divide several times before induction, it will generate all the possible derivative cells, whereas if it is isolated at the time of induction, only one type of derivative cell would be expected.

Our first evidence that substates within the stem cell compartment of PSCs might exhibit different lineage potentials came from studying the pluripotent human embryonal carcinoma cell line, NTERA2 (Tonge et al., 2010; 2011). These cells can be readily maintained as undifferentiated cells (Andrews et al., 1984), but when exposed to retinoic acid will differentiate into a variety of cell types that include neurons (Andrews 1984). However, when single cells, identified by a fluorescent marker, were monitored during differentiation, they tended to produce colonies of neurons or non-neurons, rather than mixed colonies. This suggested that prior to induction with retinoic acid, the undifferentiated cells already existed in “pro-neural” or “pro-non-neural” substates (Figure 2). If, however, the single cells were allowed to divide for 2 or 3 days before exposure to retinoic acid and then the colonies tended to be mixed, suggesting the prepatterned substates that we inferred could interconvert over this period corresponding to roughly two or three cell divisions.

To explore further the notion of lineage-biased substates, we carried out a single-cell transcriptome analysis of the SSEA3(+) and SSEA3(−) karyotypically normal and abnormal H7 human PSCs that had first led us to the notion of substates in the stem cell compartment (Gokhale et al., 2015). This indicated that some cells that express genes typical of undifferentiated cells also expressed genes pertinent to particular differentiated cells. Taking one such gene characteristic of endoderm differentiation, *GATA6*, we produced a human PSC line in which a fluorescent reporter, GFP, was inserted into the *GATA6* locus (Allison et al., 2018). A small proportion of these cells were then found to express both SSEA3 and GATA6, and these SSEA3(+)/GATA6(+) cells were similarly clonogenic to the SSEA3(+)/GATA6(−) cells. Clones isolated from both subsets regenerated similar mixed populations of SSEA3(+)/GATA6(+) and SSEA3(+)/GATA6(−) cells as the parental line, indicating that the SSEA3(+)/GATA6(+) cells were indeed still within the stem cell compartment and evidently oscillated between GATA6(+) and GATA6(−) substates. When single cells from either subset were allowed to form colonies, most of the cells within each colony expressed *OCT4*, consistent with their stem cell status, but a few cells underwent spontaneous differentiation generating cells that were OCT4(−) but expressed independent markers of endoderm differentiation, *GATA4* or *SOX17*. Notably, however, the SSEA3(+)/GATA6(+) cells generated more such endoderm-differentiated derivatives than the SSEA3(+)/GATA6(−) cells, indicating an intrinsic bias toward endoderm differentiation and consistent with the notion that the stem cell compartment comprised interconverting substates that exhibited differentiation bias. Brickman and colleagues had found similar evidence of endoderm-lineage-biased substates in mouse ES cells (Canham et al., 2010).

Using a similar approach with a human PSC line that carried a fluorescent reporter for the expression of *MIXL1*, a gene expressed early in the mesoderm lineage, we also found that the stem cell compartment included lineage-biased substates, in this case defined by differential expression of *MIXL1*, with SSEA3(+)/*MIXL1*(+) cells biased toward mesoderm differentiation (Stavish et al., 2020). We also found that by balancing cross-antagonistic external signals that tended either to promote or inhibit exit from the stem cell compartment, it was possible to maintain cells proliferating in the mesoderm-biased state, such that they could subsequently differentiate or revert to the unbiased stem cell substates if culture conditions were altered.

## An integrated experimental and modeling framework for PSC heterogeneity that reveals metastable substates

Whereas the empirical studies discussed earlier provided evidence for lineage-biased substates in PSC cultures, they provided little information about the rules governing the dynamics of these metastable substates and whether transitions between substates are purely noise driven or whether alternatives like deterministic chaos also play a role. Chaotic systems, characterized by sensitivity to initial conditions and complex trajectory evolution, can produce density distributions of states, providing a framework to explain cellular heterogeneity even in the absence of stochastic noise.

**Figure 3:**
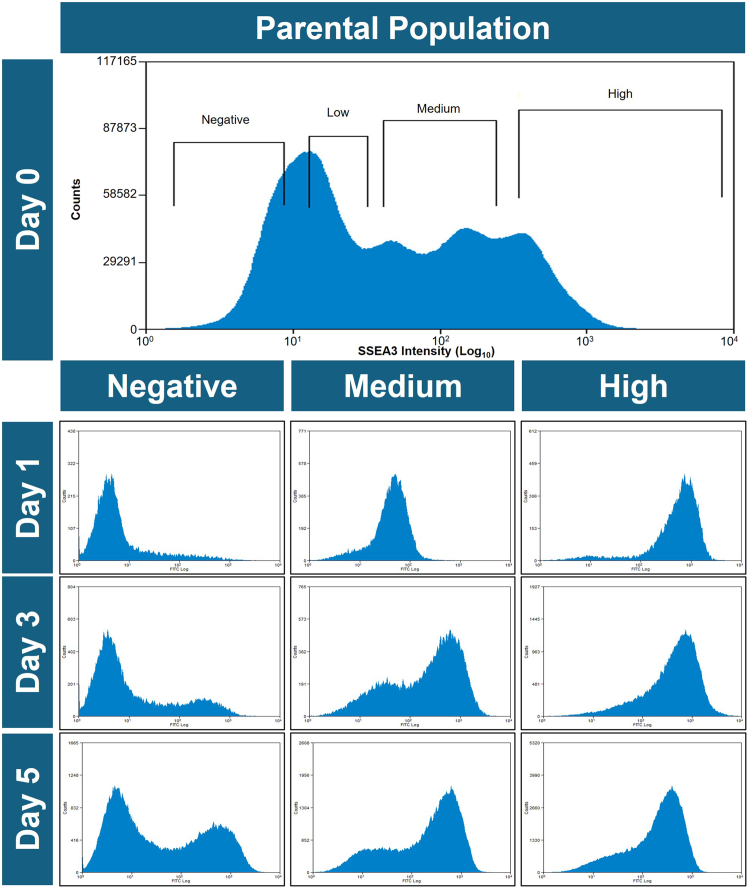
Assessment of SSEA3 expression in populations of NTERA2 pluripotent embryonal carcinoma cells by flow cytometry Subcultures of NTERA2 cells were sorted according to their SSEA3 expression levels (“negative,” “low,” “medium,” or “high”) from the parental population by fluorescence-activated cell sorting on day 0. Subcultures of each sorted fraction were then assessed daily for their SSEA3 expression over 5 days. The data shown are a subset of the complete dataset to illustrate the change of SSEA distributions from sorted fractions over time. For all fractions, there was a tendency for SSEA3 expression levels to converge toward the heterogeneous distribution as seen in the parental population, although complete convergence was not achieved within the time frame of the experiment. However, the rate of convergence to the parental distribution of SSEA3 expression varies with the expression levels from the initial sort (e.g., the “high” fraction takes longer to re-establish that distribution than the “medium” fraction).

To investigate this further, we developed a dynamical chaotic map model that predicts the evolution of subpopulations and can be used to analytically determine the presence of metastable states (Mason 2016; Nie 2015). For this, we explored the simple system based on the expression of the cell surface antigen, SSEA3, by NTERA2 cells. These cells typically express a wide spread of SSEA3 expression levels, and so, we designed an experiment to generate data to model the evolution of cells expressing different levels of SSEA3. Multiple subpopulations of cells expressing different levels of SSEA3 were isolated by fluorescence-activated cell sorting and re-cultured so that the evolution of these sorted subpopulations could be analyzed through daily measurements of SSEA3 expression over several days (Figure 3). Flow cytometry data from each fraction provided insight into the stability and variability of SSEA3 expression, allowing us to visualize how the SSEA3 expression of each fraction might develop over time. Over the measured time frame (5 days), the progeny of each fraction became increasingly heterogeneous, with populations emerging across the full range of SSEA3 expression levels as observed in the initial parental population. Although, within this time frame, the progeny of the sorted populations do not fully replicate the original SSEA3 distribution of the parental population, the distributions did tend toward the original distribution. This pattern of “reversion” toward the original distribution is consistent with the attractor hypothesis, which postulates that pluripotent cells occupy dynamic attractor states. However, other mechanisms may explain the observed reversion. The observed reversibility could reflect ergodicity-driven state exploration, which alone can drive convergence toward a unique steady-state distribution.

Flow cytometry distributions can be interpreted as probability density functions (PDFs), which mathematically represent the likelihood of cells exhibiting specific expression levels within the population. To model how the SSEA3 expression distribution in sorted NTERA2 subpopulations changed over time, we applied the methodology introduced by Nie and Coca (2015) (see supplementary material), which treats the sorted subpopulations’ SSEA3 expression distributions, measured daily, as a series of successive PDFs spaced 24 h apart (Figure 3).

**Figure 4:**
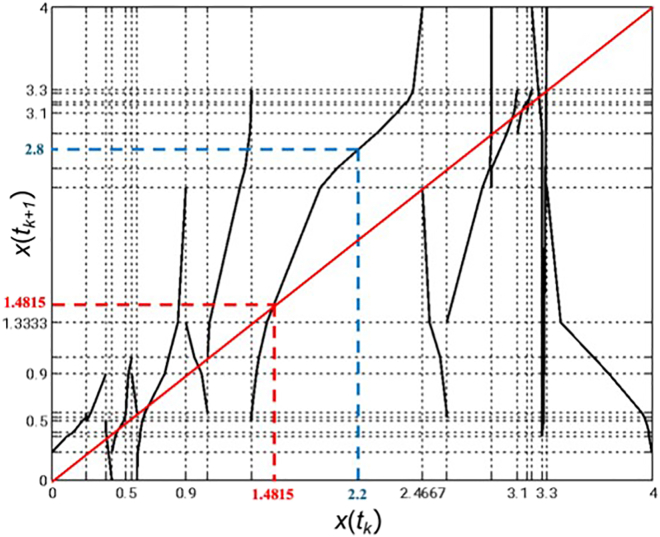
Piecewise-linear chaotic map $S\left(x\left(t_{k}\right)\right)$ of SSEA3 expression dynamics in populations of NTERA2 pluripotent embryonal carcinoma cells Axes represent the log10 SSEA3 intensity (X) on day “n” (*x* axis) and day “n+1” (*y* axis). This map represents a generalized model of SSEA3 dynamics that allows predictions of future SSEA3 intensities from present SSEA3 intensities. For example, if a cell has an SSEA3 intensity of 10^2.2^, it will be expected to have an increased SSEA3 intensity of 10^2.8^ on the following day shown by the blue dotted line. Intersections of the piecewise-linear chaotic map with the line *y* = *x* (shown in solid red) represent equilibrium points where the SSEA3 intensity of a cell is predicted not to change the following day. For example, a cell with an SSEA3 intensity of 10^1.4815^ on day “n” is anticipated to remain unchanged with respect to SSEA3 intensity on day “n+1” (dotted red line).

The flow cytometry-derived sequence of temporal PDFs, measured over a 5-day period, was used to infer a piecewise-linear chaotic map:

(Equation 4) $$x\left(t_{k+1}\right)=S\left(x\left(t_{k}\right)\right)$$

which predicts the “next-day” level of SSEA3 expression $x\left(t_{k+1}\right)$ of individual cells based on the previous day expression level $x\left(t_{k}\right)$ (Figure 4). The map was constructed using a piecewise-linear approximation, meaning that the function is defined by multiple linear segments over different intervals of SSEA3 expression. Each segment represents a different dynamic regime, with cells in certain expression ranges following distinct deterministic transitions. The boundaries between segments lead to discontinuities, where a small change in SSEA3 expression results in a sudden change in the predicted next-day expression.

The map describes how individual cells transition between expression states within the broader population, linking individual transitions to the overall dynamics of subpopulation heterogeneity. The map can be interpreted as a Markov transition process in the sense that the future state of SSEA3 expression depends only on the present state, fulfilling the Markov property. However, unlike conventional Markov chains that rely on stochastic transition matrices, the next state of the system is determined solely by the current expression level via the function S(x), without incorporating randomness or transition probabilities.

**Figure 5:**
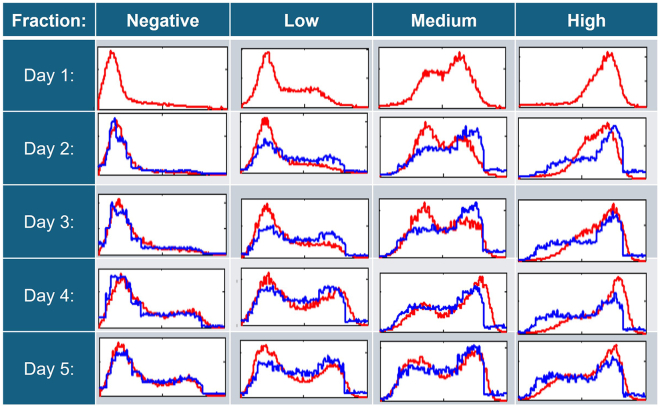
Observed (red) and predicted (blue) SSEA3 densities calculated by iterating the chaotic map $S\left(x\left(t_{k}\right)\right)$ Initial conditions used were sampled from the SSEA3 distributions measured on day 1. *x* axis; log SSEA3 intensity ranging from 10^0^ to 10^4^. The *y* axis plots the calculated probability density function transformations of the observed data: a normalization that allows cross-comparison between data (arbitrary units). The observed data (red) are compared to the predicted distributions (blue) generated using the model and the observed data from the previous day.

To generate sequences of density functions, the chaotic map model is iterated using initial conditions sampled from the PDF of the previous time step. A large sample of initial conditions is used to ensure that the resulting density functions represent the evolution of the system over time. This iterative process allows the map to simulate how subpopulations progress dynamically, with each step feeding into the next to capture transitions and reversion patterns observed in the experimental data. The model successfully predicts the dynamical evolution of any initial cell fraction toward an invariant density that matches the observed “equilibrium” distribution of SSEA3 expression of the heterogeneous NTERA-2 subpopulations (Figure 5). This convergence toward a stable density, regardless of initial conditions, is consistent with the existence of a chaotic attractor with an associated invariant measure (Lasota and Mackey, 2013). Within this stable density, individual cells continue to exhibit dynamic fluctuations, maintaining heterogeneity within the population.

The model can be used to determine analytically the unstable equilibrium points of the system by solving

(Equation 5) $$x\left(t^{*}\right)=S\left(x\left(t^{*}\right)\right)$$

The equilibrium points, which correspond to the intersection of the first diagonal with the graph of S(x) as shown in Figure 4, are potential candidates for transient but functionally relevant configurations that cells can occupy before transitioning to stable attractors during differentiation. Further isolating, tracking, and analyzing cells around the predicted equilibrium points would allow experimental validation of their transient yet functionally relevant properties.

Chaotic maps that we have used to model the evolution of SSEA3-sorted subpopulations provide a powerful framework for describing and analyzing the dynamics underpinning cellular heterogeneity. The model demonstrates how chaos can enable pluripotent cells to explore the attractor landscape, in our example collectively gravitating toward an invariant SSEA3 distribution, and provides a tool for analyzing the dynamics of stem cell substates more generally.

This framework does not exclude the role of stochastic noise, superimposed on chaotic dynamics, contributing to the maintenance of population heterogeneity while chaotic dynamics remains the dominant mechanism. An augmented methodology to infer a model that incorporates stochastic perturbations (Nie and Coca, 2018)

(Equation 6) $$x\left(t_{k+1}\right)=S\left(x\left(t_{k}\right)\right)+\eta \left(t_{k}\right)$$

can further refine this framework by accounting for the inherent noise present in biological systems. This enables more precise modeling of transitions that contribute to the maintenance of population heterogeneity. By combining deterministic chaotic maps with stochastic effects, the methodology enhances our ability to model and interpret the mechanisms that preserve heterogeneity in PSC populations.

## Conclusion

Our results and those of others imply that, removed from the constraints of the embryo, PSCs are able to oscillate between multiple metastable substates that exhibit bias in their potential fates. Knowledge of the specific GRNs that specify particular substates and the intercellular signals that control transitions between them are key to developing practical uses of PSC for regenerative medicine or other applications. They may also be pertinent to understanding the mechanisms controlling cell fate in the developing embryo.

Our model, derived from sequences of PDFs, suggests that the convergence of sorted subpopulations toward a stable distribution of SSEA3 expression, regardless of initial condition, is consistent with the presence of a chaotic attractor characterized by an invariant measure. Under steady culture conditions, the underlying GRN may operate in a chaotic regime, enabling individual cells to dynamically explore a broad range of expression states while preserving a globally stable population structure.

Within this framework, differentiation is not merely a gradual or stochastic drift away from pluripotency but may instead reflect a bifurcation, a qualitative shift in the system’s dynamics brought about by internal fluctuations or external cues. Such a transition represents a change in the system’s structural stability, leading to the emergence of a stable attractor corresponding to a lineage-committed cell fate (see supplementary material for bifurcation analysis of the reconstructed map).

A plausible mechanism for initiating this transition involves modulation of the GRN’s regulatory architecture—for example, changes in the strength, connectivity, or direction of interactions between key genes. By modulating the GRN, cells gain the flexibility required for early development while preserving the capacity to stabilize into defined lineages in response to differentiation signals. Although such modulation may involve stochastic noise, it may also follow the rules of deterministic chaos. In cancer, chaos may enable rapid adaptation of cells to therapeutic stress while also ensuring that some subpopulations are inherently resistant to therapy. These resistant subpopulations can subsequently reconstitute the tumor, contributing to relapse and progression despite initial therapeutic success.

Nevertheless, we acknowledge that similar convergence behavior could also emerge from stochastic processes. One possibility is that GRNs generate a quasi-potential landscape, within which stochastic fluctuations drive reversible transitions. Alternatively, purely ergodic stochastic frameworks describe state transitions as unbiased, noise-driven fluctuations across an unstructured landscape. While purely ergodic models can account for population-level reversion through ergodic convergence to a steady-state distribution, they lack inherent attractor structure in the transition dynamics and assume unconstrained, noise-driven exploration of state space, which may limit their ability to explain the directionality, commitment, and reproducibility of fate outcomes observed under defined experimental conditions.

Both the quasi-potential and chaotic attractor models offer mechanisms for generating metastability, heterogeneity, and steady-state convergence but differ in their underlying dynamics. In quasi-potential landscapes, differentiation is modeled as a noise-driven escape from one basin to another, whereas in chaotic systems, it reflects a bifurcation in system structure. Our findings are most consistent with the latter, although we do not exclude the possibility that stochastic fluctuations interact with deterministic structure. A hybrid model may ultimately best capture the interplay of noise, regulation, and dynamic instability that governs fate decisions in pluripotent cells. As with other dynamical systems, mathematical modeling of time series data, such as we have begun to explore with respect to substates in NTERA2 cells defined by expression of SSEA3, is necessary to provide information about the stability of different substates and the rules that govern the state transitions involved in stem cell differentiation—whether they are driven by stochastic noise or whether they represent chaotic systems.

## Acknowledgments

This work was supported by the 10.13039/501100019326UK Regenerative Medicine Platform, MRC reference grant no. MR/L012537/1, the 10.13039/501100000265Medical Research Council grant MR/V002163/1, and the European Union’s Horizon 2020 Research and Innovation program H2020-FETPROACT-2018-01 under grant agreement no. 824070. X.N. gratefully acknowledges the support from the Department of Automatic Control and Systems Engineering at the University of Sheffield and China Scholarship Council. D.C. gratefully acknowledges the support from 10.13039/501100000265MRC (grant no. G0802627), 10.13039/501100000268BBSRC (grant no. BB/M025527/1), and the Human Frontier Science Program (grant no. RGP0001/2012).

## Author contributions

The dynamical chaotic map model and its application to NTERA2 cells were carried out by J.E.M. and X.N. under the supervision of D.C.; the manuscript was largely written by D.C. and P.W.A. with contributions from J.E.M. and X.N.

## Declaration of interests

P.W.A. receives royalties from the Wistar Institute from sales of the TRA series of antibodies and is a member of the SAB of TreeFrog Therapeutics.

## References

[bib1] Allison T.F., Smith A.J.H., Anastassiadis K., Sloane-Stanley J., Biga V., Stavish D., Hackland J., Sabri S., Langerman J., Jones M. (2018). Identification and single cell functional characterization of an endodermally biased pluripotent substate in human embryonic stem cells. Stem Cell Rep..

[bib2] Andrews P.W. (1984). Retinoic acid induces neuronal differentiation of a cloned human embryonal carcinoma cell line in vitro. Dev. Biol..

[bib3] Andrews P.W., Damjanov I., Simon D., Banting G.S., Carlin C., Dracopoli N.C., Føgh J. (1984). Pluripotent embryonal carcinoma clones derived from the human teratocarcinoma cell line Tera-2. Differentiation in vivo and in vitro. Lab. Invest..

[bib4] Avery S., Zafarana G., Gokhale P.J., Andrews P.W. (2010). The role of SMAD4 in human embryonic stem cell self-renewal and stem cell fate. Stem Cell..

[bib5] Canham M.A., Sharov A.A., Ko M.S.H., Brickman J.M. (2010). Functional heterogeneity of embryonic stem cells revealed through translational amplification of an early endodermal transcript. PLoS Biol..

[bib6] Chambers I., Silva J., Colby D., Nichols J., Nijmeijer B., Robertson M., Vrana J., Jones K., Grotewold L., Smith A. (2007). Nanog safeguards pluripotency and mediates germline development. Nature.

[bib7] Chang H.H., Hemberg M., Barahona M., Ingber D.E., Huang S. (2008). Transcriptome-wide noise controls lineage choice in mammalian progenitor cells. Nature.

[bib8] Enver T., Pera M., Peterson C., Andrews P.W. (2009). Stem cell states, fates and the rules of attraction. Cell Stem Cell.

[bib9] Enver T., Soneji S., Joshi C., Brown J., Iborra F., Orntoft T., Thykjaer T., Maltby E., Smith K., Abu Dawud R. (2005). Cellular differentiation hierarchies in normal and culture adapted human embryonic stem cells. Hum. Mol. Genet..

[bib10] Fenderson B.A., Andrews P.W., Nudelman E., Clausen H., Hakomori S. (1987). Glycolipid core structure switching from globo- to lacto- and ganglio-series during retinoic acid-induced differentiation of TERA-2-derived human embryonal carcinoma cells. Dev. Biol..

[bib11] Gokhale P.J., Au-Young J.K., Dadi S., Keys D.N., Harrison N.J., Jones M., Soneji S., Enver T., Sherlock J.K., Andrews P.W. (2015). Culture adaptation alters transcriptional hierarchies among single human embryonic stem cells reflecting altered patterns of differentiation. PLoS One.

[bib12] Goodwin B.C. (1963).

[bib13] Huang S., Eichler G., Bar-Yam Y., Ingber D.E. (2005). Cell fates as high-dimensional attractor states of a complex gene regulatory network. Phys. Rev. Lett..

[bib14] Huang S. (2009). Reprogramming cell fates: reconciling rarity with robustness. Bioessays.

[bib15] Furusawa C., Kaneko K. (2009). Chaotic expression dynamics implies pluripotency: when theory and experiment meet. Biol. Direct.

[bib16] Kauffman S.A. (1969). Metabolic stability and epigenesis in randomly constructed genetic nets. J. Theor. Biol..

[bib17] Kauffman S.A. (1993).

[bib18] Laslett A.L., Grimmond S., Gardiner B., Stamp L., Lin A., Hawes S.M., Wormald S., Nikolic-Paterson D., Haylock D., Pera M.F. (2007). Transcriptional analysis of early lineage commitment in human embryonic stem cells. BMC Dev. Biol..

[bib19] Lasota A., Mackey M.C. (2013).

[bib20] Mason J.E. (2016).

[bib21] Nie X., Coca D. (2015). Reconstruction of one-dimensional chaotic maps from sequences of probability density functions. Nonlinear Dyn..

[bib22] Nie X. (2015).

[bib23] Nie X., Coca D. (2018). A matrix-based approach to solving the inverse Frobenius–Perron problem using sequences of density functions of stochastically perturbed dynamical systems. Commun. Nonlinear Sci. Numer. Simul..

[bib24] Prager B.C., Bhargava S., Mahadev V., Hubert C.G., Rich J.N. (2020). Glioblastoma stem cells: driving resilience through chaos. Trends Cancer.

[bib25] Smith A. (2024). Propagating pluripotency – The conundrum of self-renewal. Bioessays.

[bib26] Stavish D., Böiers C., Price C., Frith T.J.R., Halliwell J., Saldaña-Guerrero I., Wray J., Brown J., Carr J., James C. (2020). Generation and trapping of a mesoderm biased state of human pluripotency. Nat. Commun..

[bib27] Tonge P.D., Olariu V., Coca D., Kadirkamanathan V., Burrell K.E., Billings S.A., Andrews P.W. (2010). Prepatterning in the stem cell compartment. PLoS One.

[bib28] Tonge P.D., Shigeta M., Schroeder T., Andrews P.W. (2011). Functionally defined substates within the human embryonic stem cell compartment. Stem Cell Res..

[bib29] Waddington C.H. (1957).

[bib30] Zhou J.X., Aliyu M.D.S., Aurell E., Huang S. (2012). Quasi-potential landscape in complex multi-stable systems. J. R. Soc. Interface.

